# Impact of cesarean section on timely initiation of breastfeeding in Ethiopia: a systematic review and meta-analysis

**DOI:** 10.1186/s13006-021-00399-9

**Published:** 2021-07-05

**Authors:** Temesgen Getaneh, Ayenew Negesse, Getenet Dessie, Melaku Desta, Habtamu Temesgen, Tadesse Getu, Kihinetu Gelaye

**Affiliations:** 1 Department of Midwifery, College of Health Science, Debre Markos University, Debre Markos, Ethiopia; 2 Department of Human Nutrition and Food Sciences, College of Health Science, Debre Markos University, Debre Markos, Ethiopia; 3 Center of Excellence in Human Nutrition, School of Human Nutrition, Food Science and Technology, Hawassa University, Awasa, Ethiopia; 4 Department of Nursing, School of Health Science, College of Medicine and Health Science, Bahr Dar University, Bahir Dar, Ethiopia; 5 Department of Midwifery, Hosanna Health Science College, Hosanna, Ethiopia; 6 Department of Midwifery, School of Health Science, College of Medicine and Health Science, Bahr Dar University, Bahir Dar, Ethiopia

**Keywords:** Timely initiation of breastfeeding, Cesarean section, Ethiopia, Systematic review and meta-analysis

## Abstract

**Background:**

Timely initiation of breastfeeding is feeding of breast milk within one hour of birth, however, three in five babies were not breastfed in the first hour of birth globally. There is evidence that cesarean section is the major constraint for this low prevalence, but the impact of cesarean section on timely initiation of breastfeeding in Ethiopia is limited. Therefore, this meta-analysis aimed to provide evidence for policy makers, health professionals and program implementers.

**Methods:**

This systematic review followed the Preferred Reporting Items for Systematic reviews and Meta-Analysis guidelines. Electronic bibliographic databases such as PubMed/Medline, EMBASE, PsycINFO, CINHAL, Scopus, Google Scholar, Science Direct and Cochrane Library were used to search relevant studies and was conducted up to February 2021. Random effects model meta-analysis was applied to estimate the pooled impact of cesarean section on timely initiation of breastfeeding with 95% confidence intervals (CI). I^2^ statistical test and, funnel plot and Egger’s test were used to check heterogeneity and publication bias across included studies respectively.

**Results:**

According to meta-analysis of 17 studies, the pooled estimate of timely initiation of breastfeeding among women who had cesarean section in Ethiopia was 40.1% (95% CI 33.29, 46.92). The meta-analysis of 29,919 study participants showed that cesarean section was associated with a 79% lower odds of timely initiation of breastfeeding compared with vaginal birth (OR 0.21; 95% CI 0.16, 0.28).

**Conclusions:**

In Ethiopia, almost only one-third of mothers who gave birth by cesarean section initiate breastfeeding within one hour of birth, much lower than the pooled prevalence among general population. Special health promotion, intervention and healthcare provider support during immediate or early skin to skin contact, and having focused breastfeeding guidelines for post-operative patient and trained health professionals should be considered for mothers who give birth through cesarean section.

**Supplementary Information:**

The online version contains supplementary material available at 10.1186/s13006-021-00399-9.

## Background

Timely initiation of breastfeeding is one of the most effective ways to ensure infant health and survival [1]. World Health Organization (WHO) and United Nation International Children Emergency Fund recommended breast milk as an ideal food and the exclusive nutrient source for infants for the first six months of life, and that breastfeeding should be initiated within the first hour of birth [1, 2]. Breastfeeding is associated with direct short and long term benefits for both the infant and the mother [3]. Evidence from developing countries showed that the overall morbidity such as hospitalization and rate of acute illness and mortality were much lower among infants who initiated timely breastfeeding [4, 5]. It has also familial and societal benefits in terms of reduction in expenses of infant formula fed and hospitalization [6].

Despite this paramount short- and long-term advantages of timely initiated breastfeeding, it is still unacceptably low especially in developing countries including our country Ethiopia. Globally, three in five babies were not breastfed within the first hour of birth [1]. A meta-analysis held in Middle East countries revealed that only 34.3% of neonates received breast milk within one hour [7]. Maternal sociodemographic variables include; educational status, employment status, and obstetric interventions like cesarean section were factors influencing timely initiation of breastfeeding [7–9]. As the global rate of cesarean section was increased, its impact on timely initiation of breastfeeding became a major concern [10, 11]. Evidence from secondary analysis of WHO global survey showed that only 39.7% of infants delivered through cesarean section initiated breastfeeding within one hour of birth [11].

A systematic review and meta-analysis of 54 studies from 33 countries confirmed that early initiation of breastfeeding was lower after cesarean section when compared with vaginal delivery (pooled OR 0.57; 95% CI 0.50, 0.64) [12]. Similarly, evidence from 33 Sub-Sahara African countries showed that cesarean section was associated with a 46% reduction in timely initiation of breastfeeding [13]. Furthermore, studies from Bangladesh, Canada and China supported that women who had cesarean section were less likely to initiate breastfeeding early after birth when compared with women who had a vaginal birth [14–16]. This lower rate of timely initiation of breastfeeding associated with cesarean section might be related to physical separation of infant and mother during recovery time, anesthesia effect, restricted mobility, and distressful condition of neonate and critical condition of the mother after cesarean delivery [9, 17]. In general, cesarean sections have negative impact on maternal physical, physiological and psychological responses, and affect timely initiation of breastfeeding.

However, the insight given to the impact of cesarean section on timely initiation of breastfeeding in Ethiopia is limited. There are inconsistent studies conducted in Ethiopia reported variable findings [18–26], even though that it was not the primary outcomes of most studies. Therefore, this is the first meta-analysis done to explore the impact of cesarean section on timely initiation of breastfeeding in Ethiopia, providing timely and relevant policy and administrative information and national based estimates. Identifying and addressing factors that impede early initiation of breastfeeding is important for healthcare professionals to provide evidence-based education and support, and to improve the breastfeeding rate. So that, it will reduce neonatal morbidity and mortality.

## Methods

### Searching strategy

Searching of relevant published literature was performed using electronic bibliographic databases; PubMed/Medline, EMBASE, PsycINFO, CINHAL, Scopus, Google Scholar, Science Direct and Cochrane Library. This systematic search was undertaken till February/2021. The reference lists of all identified articles and local institutional websites were checked for additional sources. The overall searching method was conducted using keywords including timely initiation of breastfeeding OR early initiation of breastfeeding OR delay initiation of breastfeeding OR initiation of breastfeeding AND cesarean section OR cesarean delivery AND Ethiopia and the medical searching heading (MeSH) terms for each selected keywords. Searching terms were combined using “OR and AND” Boolean operators. This systematic review followed the Preferred Reporting Items for Systematic Reviews and Meta-Analyses Protocols (PRISMA) checklist guidelines [27] (Additional file 1).

### Selection of studies

Two reviewers (TG and GD) assessed titles and abstracts of all identified studies. Then, full texts of potentially eligible studies in the first review were assessed independently again for inclusion. Any disagreement between reviewers were solved by discussion, and if not the third reviewer (AN) was engaged.

### Inclusion and exclusion criteria

In general, studies included in this meta-analysis were 1) all observational studies (the available), 2) both published and gray literature, 3) studies reported in English language, 4) studies conducted on mothers who gave birth through cesarean section and reporting timely initiation of breastfeeding and 5) studies available till February/2021. Whereas, studies excluded in this review were; 1) studies with poor quality, 2) case reports, reviews and unable to access their full texts (after two email requests to authors) were excluded from this review and 3) studies which didn’t report timely initiation of breastfeeding. In general, no restriction was stated in terms of study year, publication, study design and setting.

### Outcome of interest

The outcomes of this systematic review and meta-analysis were to estimate the pooled prevalence of timely initiation of breastfeeding among women who gave birth by cesarean section and to explore the pooled impact of cesarean section on timely initiation of breastfeeding in Ethiopia. Timely initiation of breastfeeding is defined as the start of breast milk feeding or putting of the newborn on the breast to fed milk within one hour of birth [28]. Log odds ratio was used to estimate its effect size.

### Data extraction

Standardized Microsoft Excel spreadsheet prepared format was used to extract relevant data from the included primary studies. The following data were extracted by two reviewers independently; first authors name, publication year, geographical setting, study design, study setting (facility or community based), sampling technique, sample size, response rate, prevalence and two by two table to assess the effect of cesarean section on timely initiation of breastfeeding.

### Methodological quality assessment

The quality of primary studies was evaluated using Joanna Briggs Institute (JBI) critical quality appraisal tool for prevalence studies [29]. The tool included a total of eight criteria: 1) sampling and design, 2) sampling frame, 3) sample size, 4) measurement tool, 5) outcome measurement, 6) response rate, 7) confidence interval and subgroup analysis and 8) study subjects. Two reviewers (TG and AN) assessed each studies independently. Then, studies with score of five and above out of eight were considered as low risk and included in this review. Any disagreement between reviewers was resolved through discussion and consensus, if not the third reviewer (MD) was involved (Additional file 2).

### Data analysis

The result of this meta-analysis was analyzed using STATA version 15 software (Stata Corp, College Station, Texas, USA). Heterogeneity across primary studies was checked using standard I^2^ statistical test. Random effect model was computed to estimate Der Simonian and Laird’s pooled effect of cesarean section on timely initiation of breastfeeding, since I^2^ revealed a significant heterogeneity (I^2^ = 93.2% and *p* = 0.000). In addition, Egger’s statistical test and visual inspection of funnel plot were used to examine publication bias. Accordingly, there was no publication bias as evidenced with Egger’s test *p* = 0.33 and relatively symmetrical distribution of funnel plot. Furthermore, subgroup analysis was also conducted based on geographical setting, publication status, study setting and response rate to minimize random variation between their point estimates. Forest plot was used to present results of this meta-analysis. Odds Ratio (OR) with 95% Confidence Interval (95% CI) was also determined.

## Results

### Characteristics of included studies

**Fig. 1:**
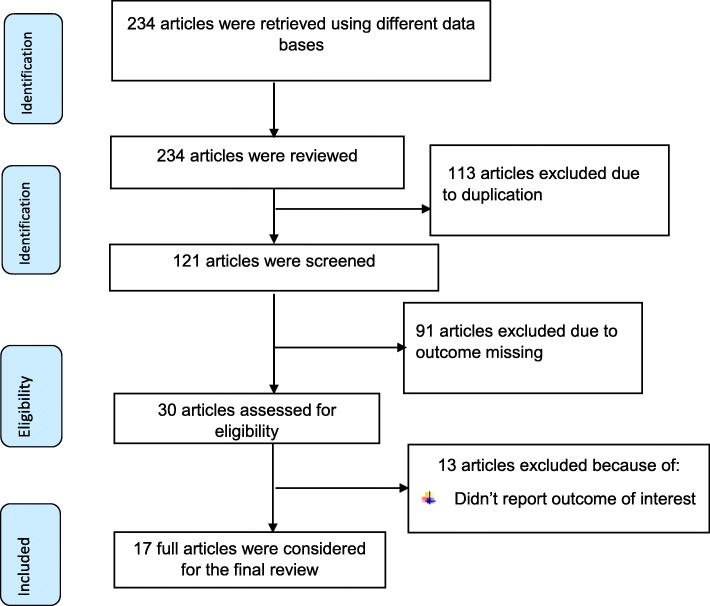
PRISMA flow diagram of included studies to estimate the pooled impact of timely initiation of breastfeeding in Ethiopia

Of the total of 234 studies retrieved from different data bases; 121 studies were remained after removing of 113 duplicate articles. Then, additional 91 studies were excluded because they didn’t meet the inclusion criteria. After full texts of 30 studies assessed, 13 studies were excluded because of didn’t report outcome of interest. Finally, 17 primary studies were included to estimate the pooled impact of cesarean section on timely initiation of breastfeeding in Ethiopia (Fig. 1).

Among the included studies, seven of them were from Amhara region [19, 20, 24, 25, 30–32], two studies from each of the following; Tigray [22, 33], Oromo [34, 35], South Nation Nationality and Peoples region (SNNPR) [18, 36] and Ethiopian Demographic Health Survey (EDHS) data [21, 26] while the remaining two studies were contributed from Addis Ababa [37] and Afar region [38] (one study for each). All eligible studies in this review were cross-sectional study design consisting 29,919 mothers as study participant. Regarding to publication status, nearly 90% were published from 2011 to 2020 in international journals (15 studies). In Ethiopia, a minimum of 20% [25] to a maximum of 71.4% [24] mothers who gave birth through cesarean section initiate breastfed within one hour of birth. Table 1 summarizes the characteristics of included studies.

**Table 1:** Descriptive summary of included studies in this review of impact of cesarean section on timely initiation of breastfeeding in Ethiopia

Author	Study Year	Region	Study design	Setting	Technique	Response rate (%)	Sample size	Prevalence (%)	Cesarean section OR (95% CI)	JBI
Ahmed et al. [26]	2016	EDHS	Cross-sectional	Community	Cluster	100	336	27	0.24 (0.19, 0.31)	6
Ayalew et al. [25]	2016	Amhara	Cross-sectional	Community	SRS	95	71	20	0.08 (0.04, 0.16)	5
Belachew [24]	2017	Amhara	Cross-sectional	Community	SRS	94.5	185	71.4	0.69 (0.45, 1.05)	6
Berhe et al. [33]	2011	Tigray	Cross-sectional	Facility	SRS	100	24	33.3	0.08 (0.03, 0.19)	7
Dejen [37]	2020	Addis Ababa	Cross-sectional	Facility	Consecutive	100	413	48.2	–	6
Gargamo et al. [36]	2019	SNNPR	Cross-sectional	Community	Cluster	97.7	50	42	0.14 (0.08, 0.27)	5
Gebremeskel et al. [22]	2018	Tigray	Cross-sectional	Community	SRS	99.2	28	35.8	0.33 (0.15, 0.72)	5
Gedefaw et al. [21]	2016	EDHS	Cross-sectional	Community	Cluster	100	165	37.8	0.20 (0.15, 0.28)	5
Getnet et al. [32]	2020	Amhara	Cross-sectional	Facility	SRS	98	356	51.9	0.21 (0.11, 0.41)	7
Liben et al. [38]	2015	Afar	Cross-sectional	Community	SRS	99	55	22.6	0.45 (0.23, 0.89)	5
Seid et al. [20]	2012	Amhara	Cross-sectional	Community	Cluster	100	91	63.7	0.19 (0.12, 0.32)	5
Setegn et al. [34]	2010	Oromo	Cross-sectional	Community	Cluster	91	18	33.3	0.43 (0.16, 1.17)	6
Shiferaw et al. [19]	2017	Amhara	Cross-sectional	Facility	SYRS	100	421	57	–	6
Tewabe et al. [31]	2015	Amhara	Cross-sectional	Community	SRS	95.7	38	42.1	0.13 (0.06, 0.25)	7
Tilahun et al. [30]	2013	Amhara	Cross-sectional	Community	stratified	98.3	29	20.7	0.14 (0.05, 0.34)	5
Woldemicheal et al. [35]	2014	Oromo	Cross-sectional	Community	SRS	96.6	18	33.3	0.22 (0.08, 0.61)	5
Yohannes et al. [18]	2019	SNNPR	Cross-sectional	Community	SRS	100	29	44.8	0.16 (0.07, 0.35)	5

Note: SRS-simple random sampling; SYRS-systematic random sampling; JBI- Joanna Briggs Institute critical quality appraisal tool

### Pooled prevalence of timely initiation of breastfeeding in Ethiopia

**Fig. 2:**
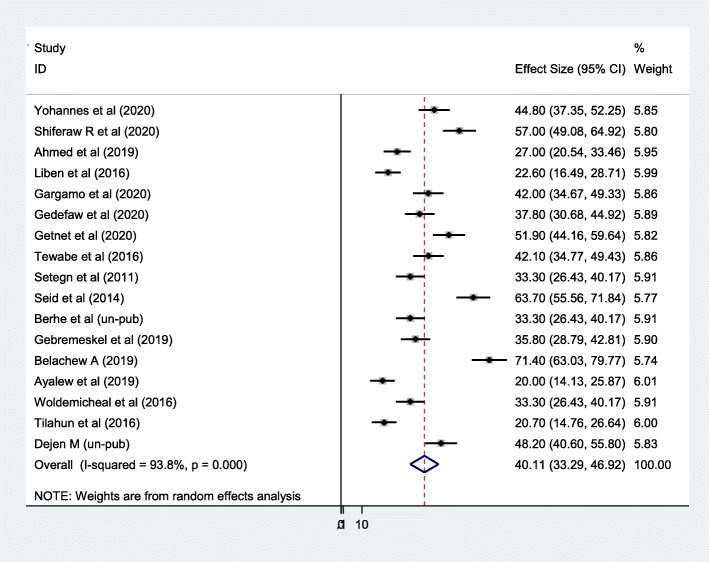
Forest plot of pooled estimate of timely initiation of breastfeeding after cesarean section in Ethiopia

**Fig. 3:**
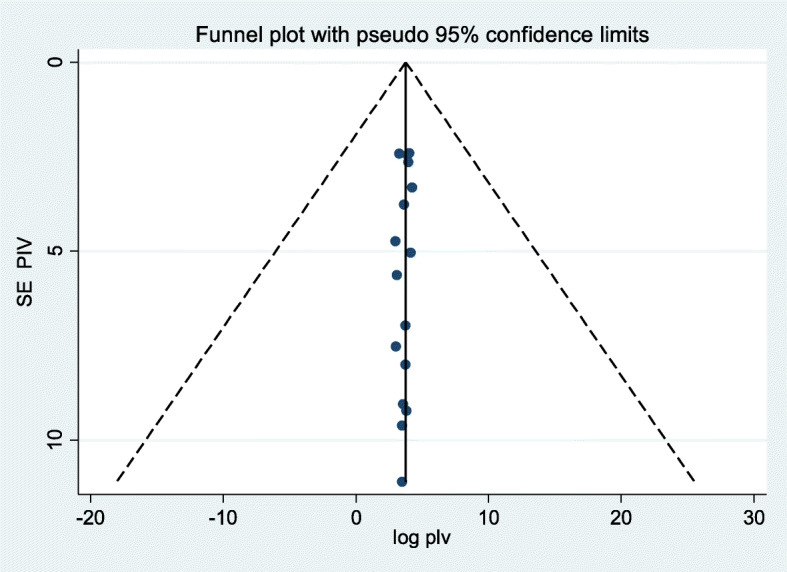
Funnel plot to show symmetrical distribution of included studies

According to the pooled random effect estimate of 17 studies, only 40.1% of mothers who gave birth by cesarean section were able to initiate breastfeeding within one hour of birth in Ethiopia (95% CI 33.29, 46.92), i.e., 59.9% of infants were not put to the breast within one hour of birth (Fig. 2). From this systematic review, a significant heterogeneity was observed across included primary studies, evidenced with I^2^ = 93.8%, *p* < 0.001. However, non-significant Egger’s statistical test (*p* = 0.33) and relatively symmetrical visualization of funnel plot (Fig. 3) excluded the presence of possible publication bias. Subgroup analysis was also conducted based on different study characters as indicated below in Table 2.

**Table 2:** Sub group analysis of impact of cesarean section on timely initiation of breastfeeding with different study characteristics in Ethiopia

Subgroup	Characteristic	No. of studies	Prevalence (95% CI)	Heterogeneity statistics	I^**2**^	***p***-value
Region	Amhara	7	46.5 (32,60)	197.8	97	< 0.001
	SNNPR	2	43.3(38,48)	0.28	0.00	0.599
	Tigray	2	34.5 (29,39)	0.25	0.00	0.618
	Oromo	2	33.3 (28,38)	0.00	0.00	1.00
	EDHS	2	32.2 (21, 42)	2.85	79.4	0.028
	Others	2	35.2 (10,60)	26.49	96.2	< 0.001
Publication	Published	15	40 (32,47)	246.8	94.3	< 0.001
	Unpublished	2	40 (33,46)	8.13	87.7	0.004
Setting	Community based	13	37.8 (29, 45)	204.3	94.1	< 0.001
	Facility based	4	47.4 (37,57)	22.98	86.9	< 0.001
Response rate	> 97%	11	40 (31,48)	153	93.5	< 0.001
	< 98%	8	40 (27,53)	103	95.1	< 0.001

### Impact of cesarean section on timely initiation of breastfeeding in Ethiopia

**Fig. 4:**
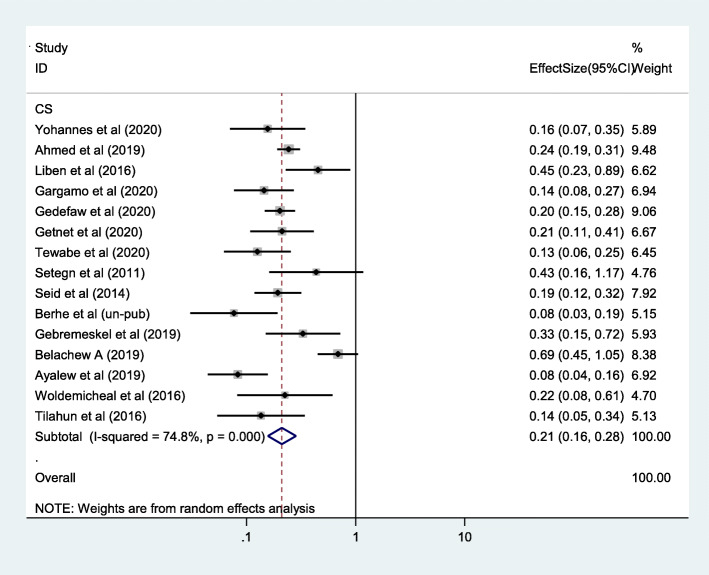
Forest plot of the impact of cesarean section on timely initiation breastfeeding in Ethiopia

The main aim of this meta-analysis was to estimate the pooled impact of cesarean section on timely initiation of breastfeeding. Accordingly, the analysis of 15 primary studies showed that cesarean section was associated with 79% lower prevalence of timely initiation of breastfeeding in Ethiopia (OR 0.21, 95% CI 0.16, 0.28) (Fig. 4). No publication bias was observed as evidenced with Egger’s statistical test with *p*-value of 0.41.

## Discussion

Generally, this systematic review and meta-analysis is the first review conducted in Ethiopia aimed at estimating the consolidated impact of cesarean section on timely initiation of breastfeeding. Even though WHO recommended that exclusive breastfeeding should be initiated within one hour of birth for all infants, more than half of infants in developing countries did not initiate breastfeeding in a timely manner. There is some evidence that Cesarean section is the most common constraining factor [9, 39–42]. Our country, Ethiopia is one of these developing countries striving to reduce unacceptably high neonatal morbidity and mortality, in which delayed initiation of breastfeeding was the main contributor. Therefore, conducting such concrete scientific evidence in Ethiopia provides a baseline for health policy makers and program implementers to reduce neonatal mortality and to improve both maternal and infant health.

The present meta-analysis showed that almost only one-third of infants born with cesarean section initiated exclusive breastfeeding within one hour of birth (40.1, 95% CI 33.29, 46.92%), whereas 72.4% of mothers who gave birth vaginally initiated breastfeeding within one hour of birth. This finding is in line with secondary analysis of WHO global survey, which reported that 39.7% of women who had cesarean delivery start to breastfeed to their infant within one hour of birth [11]. A study conducted in China was also support our finding [15]. The pooled prevalence of timely initiation of breastfeeding among mothers who gave birth by cesarean section estimated in this meta-analysis was much lower than the overall pooled prevalence of timely initiation of breastfeeding among the total population in Ethiopia and secondary analysis of WHO global survey, which was 61 and 57.6% respectively [11, 43]. In addition, higher prevalence of timely initiated breastfeeding after cesarean section was reported in India [44], and Malawi [45]. However, the present finding was higher than findings of studies conducted in Egypt [46], India [47], Bangladesh [14] and Uganda [48]. The possible explanation might be due to variation in sociodemographic characteristics of the population, differences in health coverage including antenatal care and institutional delivery service, incidence of cesarean section and governmental concern [49, 50].

In regarding to regional prevalence, 46% of infants born by cesarean section initiated breastfeeds within one hour of birth in Amhara regional state. This could be due to regional variation in antenatal care and institutional delivery coverage services [49]. In regarding publication status, there was no difference observed between published and un-published primary studies. However, higher percentage of infants born by cesarean section were initiated breastfed earlier among studies conducted on facility based (47%). The possible explanation might be giving birth at institution has further advantage in terms of healthcare giver counseling and support for breastfeeding.

In addition to this low pooled prevalence of timely initiation of breastfeeding, this meta-analysis also evidenced that cesarean section significantly decreased rate of timely initiation of breastfeeding when compared with vaginal delivery. Accordingly, cesarean section was associated with 79% lower the prevalence of timely initiation of breastfeeding in Ethiopia (OR 0.21, 95% CI 0.16, 0.28%). This finding is consistent with result of meta-analysis done on 54 studies among 33 countries, which reported that early breastfeeding initiation was lower after cesarean delivery compared with vaginal delivery (OR 0.57, 95% CI 0.50, 0.64) [12]. Similarly, a meta-analysis done on 33 Sub Saharan African countries showed that cesarean section was associated with 46% lower prevalence of timely initiation of breastfeeding [13]. All these findings were also supported by findings of studies done in India [44], Australia [9], Canada [16], Bangladesh [14], Malawi [45] and Uganda [48]. This significant association of cesarean section and timely initiation of breastfeeding might be due to limited postoperative mobility, postoperative pain, and ongoing management of medical complications [9, 12]. In adding to this, critical condition of the infant and admission to neonatal intensive care unit (as most cesarean section is performed due to obstetric complications like fetal distress, antepartum hemorrhage), and effect of anesthesia (especially if is general anesthesia) might have also their own effect [17]. Therefore, doing cesarean section based on appropriate indication is important to decrease the rate of unnecessary cesarean section especially in developing countries. In turn, this will have an effect in decreasing cesarean section negative impact on timely initiation of breastfeeding. In addition, healthcare provider support is important in post operation recovery room especially to support and advocate early skin to skin contact [51, 52]. So, additional staff to assist early breastfeeding initiation and effective pain management is very important.

### Limitation of the study

Finally, the present systematic review and meta-analysis is not without limitation. Thus, all included studies were cross-sectional study design, there might be difficult to control confounding variables. This meta-analysis didn’t include all regional state of Ethiopia, as only six were included. There are also studies having small sample size. Finally, the forest plot indicates presence of significant heterogeneity across primary studies.

## Conclusions

Generally, almost only one-third of mothers (40%) who had cesarean section initiated breastfeeding within one hour of birth, which is much lower than pooled prevalence of timely initiation of breastfeeding among general population in Ethiopia and far from WHO nutrition target 2025. In addition, cesarean section was associated with a 79% lower odds of timely breastfeeding initiation compared with vaginal birth. Even though, early initiation of breastfeeding within one hour of birth for all infants regardless of mode of delivery is needed, special health promotion like parent education, health professional intervention and support like immediate or early skin to skin contact should be considered for mothers who give birth through cesarean section. In addition, performing a cesarean section according to the WHO recommended rate is important.

## Supplementary Information

**Additional file 1.** Preferred Reporting Items for Systematic Reviews and Meta-Analyses Protocols (PRISMA) checklist guidelines

**Additional file 2.** Joanna Briggs Institute (JBI) critical quality appraisal tool for prevalence studies

## Data Availability

Data will be available from the corresponding author upon reasonable request.

## Abbreviations

CI: Confidence Interval
EDHS: Ethiopian Demographic Health Survey JBI-Joanna Briggs Institute
OR: Odds Ratio
PRISMA: Preferred Reporting Items for Systematic Reviews and Meta-Analyses Protocols
SNNPR: South Nation Nationality and Peoples Region
WHO: World Health Organization
